# Difficult fetal extraction in cesarean section: Number 8 – 2024

**DOI:** 10.61622/rbgo/2024FPS08

**Published:** 2024-08-15

**Authors:** Álvaro Luiz Lage Alves, Alexandre Massao Nozaki, Lucas Barbosa da Silva

**Affiliations:** Universidade Federal de Minas Gerais Hospital das Clínicas Belo Horizonte MG Brazil Hospital das Clínicas, Universidade Federal de Minas Gerais, Belo Horizonte, MG, Brazil.; Universidade de São Paulo Faculdade de Medicina Hospital das Clínicas São Paulo SP Brazil Hospital das Clínicas, Faculdade de Medicina da Universidade de São Paulo, São Paulo, SP, Brazil.; Hospital das Clínicas São Sebastião SP Brazil Hospital das Clínicas, São Sebastião, SP, Brazil.

## Abstract

The main causes of difficult fetal extraction during cesarean section are deeply impacted fetal head and floating presentation of the fetus.

Studies of management techniques for difficult fetal extraction during cesarean section and the maternal and neonatal results lack scientific evidence, as these predominantly come from case reports, small case series and expert opinions.

The deeply impacted fetal head is usually associated with prolongation of the expulsion period and/or unsuccessful attempts at operative vaginal delivery.

The main maternal complications associated with the management of the deeply impacted fetal head are lacerations in the lower uterine segment, hematomas in the uterine ligaments and injuries to the uterine vessels, cervix and/or urinary tract.

The main neonatal complications associated with the management of a deeply impacted fetal head are intracranial hemorrhage, fractures of the skull and/or cervical spine, nerve injuries, perinatal asphyxia and even death.

Among the maneuvers for delivery of the deeply impacted fetal head, the abdominovaginal delivery (push method) seems to be the most associated with maternal and neonatal complications.

In the non-insinuated and floating fetal head, the internal podalic version followed by pelvic extraction differs from the reverse breech extraction (pull method). When the fetal head is high in the pelvis, the fetus is internally ejected before the extraction of its body segments, similar to the internal version performed in the vaginal delivery of the second twin with floating presentation of the fetus.

## Recommendations

- The main strategies for obtaining atraumatic fetal extractions in cesarean sections are the creation of appropriately sized incisions and the institution of pharmacological body and uterine relaxation.
- Repairing full-thickness defects of the myometrium and performing hysterotomies in the upper zone of the uterine segment apparently contributes to eliminate the risk of the placenta accreta spectrum (PAS) associated with low hysterotomies performed in advanced stages of labor and when fetal extraction is difficult.
- Preparation for the management of the deeply impacted fetal head must include guidance for the parturient woman and her companions regarding obstetric challenges, discussion between the obstetrics, anesthesiology and nursing teams, and the development of a fetal extraction plan. Uterine relaxation and good positioning of the hysterotomy are recommended. The hand that will manipulate the fetal head must be slowly and carefully inserted. In abdominovaginal delivery, the woman must be positioned with her lower limbs elevated, and the time after hysterotomy must be timed, recorded and communicated.
- In the management of the deeply impacted fetal head, very low hysterotomies should be avoided. One should not act with haste and force, and a few seconds should be spent evaluating the anatomy, even in the presence of fetal bradycardia. When manipulating the fetal head, the operator must not flex the wrist against the myometrium, between the incision and the uterine cervix.
- The main maneuvers for the abdominal release of deeply impacted fetuses are the abdominovaginal delivery (push method), the reverse breech extraction (pull method) and the Patwardhan maneuvers.
- In abdominovaginal delivery, the associated use of obstetric levers (or a branch of forceps) or disimpacting systems ("fetal pillow") potentially reduces the likelihood of uterine trauma and other complications.
- In the non-insinuated and floating fetal head, the internal podalic version followed by pelvic extraction or extraction with the aid of the vacuum extractor, lever or forceps are the easiest and safest options; the first option is usually faster.

## Background

Despite its low incidence, difficult fetal extraction during cesarean section is an eventuality associated with increased maternal and neonatal morbidity. Among the various causes that make fetal extraction in cesarean sections difficult, the most notable are the deeply impacted fetal head and floating presentation of the fetus.^(1)^

At the same time, an increase in the rates of cesarean sections performed in the second stage of labor has been observed, often due to failure or lack of attempt at operative vaginal birth.^(2)^ In addition, the increase in the prevalence of high-risk pregnancies that motivate earlier terminations favors the occurrence of other factors complicating fetal extraction, with emphasis on low birth weight and anomalous presentations.^(3)^

Although there have been advances in studies related to techniques for managing difficult vaginal births, the information available regarding difficulties in cesarean sections lacks scientific evidence, which predominantly comes from case reports, small case series and expert opinions. Therefore, training obstetricians in related skills and investing in studies with more robust levels of evidence and degrees of recommendations are essential actions to optimize the management of difficult fetal extraction during cesarean section.^(4)^

## What are the main causes of difficult fetal extraction in cesarean sections and the main associated factors?

Deeply impacted fetal head and floating fetal presentation are the main causes of difficult fetal extraction during cesarean section. Other causes include extremely low birth weight fetuses, breech and transversal presentations, placentas implanted in the anterior segment, uterine leiomyomas and the presence of Bandl's ring in cases of imminent uterine rupture.^(1)^

Cases of deeply impacted fetal head are often accompanied by prolongation of the expulsion period and/or failed attempts at operative vaginal delivery. Floating presentations of the fetus are associated with cesarean sections performed in the absence of labor and changes in fetal statics (transverse and oblique situations).^(1,4)^

## What is the association between difficult fetal extraction during cesarean section and the placenta accreta spectrum?

Undoubtedly, uterine trauma is the main causal factor of the PAS. Therefore, cesarean section is the main risk factor. The healing process of hysterotomies, through fibrosis, creates tissue without elasticity. When pulled, this tissue is predisposed to additional damage, myometrial thinning, dehiscence, uterine defects and collagen exposure, all associated with the etiology of PAS. This pathophysiological mechanism explains the association of PAS with multiple cesarean sections and other causes of uterine damage, such as uterine dilation and curettage.^(5)^

The occurrence of severely invasive placentas in patients after the first cesarean section and in the absence of other causal factors raises the possibility of other mechanisms producing a primary uterine defect. Extensive uterine defects occurring in patients operated after the advanced dilation phase and/or during the expulsion period have already been demonstrated. In these situations, the hysterotomy is usually performed a few centimeters from the internal cervical os and there is intense action of collagenase in the lower uterine segment, resulting in anatomical changes. Subsequent healing with a full-thickness defect favors spontaneous lower uterine dehiscence.^(6)^

Low transverse hysterotomies potentially compromise irrigation from the cervicouterine arteries and promote areas of hypovascularization in the low uterine segment. In these areas, the absence of decidual development below the uterine incision is also observed in subsequent pregnancies, with only the amnion present in these thin regions.^(7)^ Histologically, the segment integrates the uterine cervix and presents a high percentage of collagen, which is progressively greater in areas close to the internal cervical os. Therefore, primary repair of low-segment full-thickness defects has a high rate of spontaneous recurrence, unlike more superior segmental areas. In cases of spontaneous uterine dehiscence, immunocytochemical analyzes of the edge defects reveal a complete absence of growth factors, explaining the tissue rupture and the high rate of recurrence after primary repair.^(5)^

**Figure 1:**
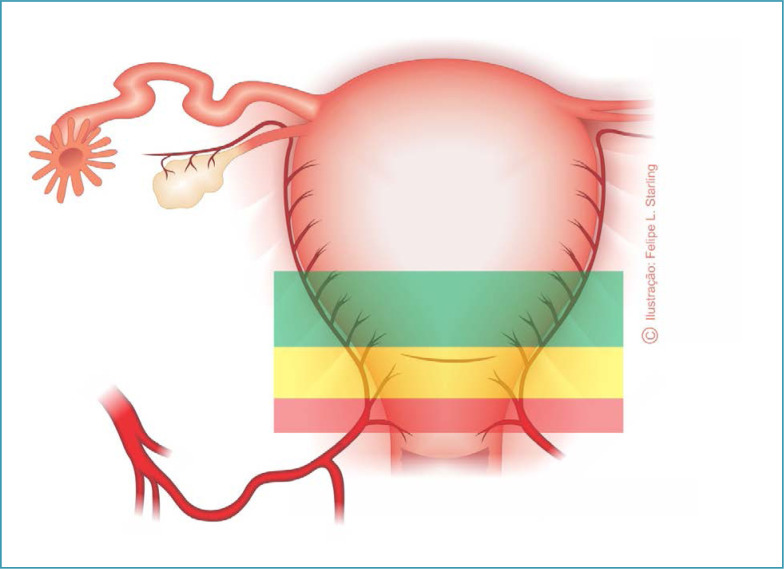
Topographic representation of uterine segment areas according to collagen percentage and blood supply

Source: Illustration by Felipe Lage Starling (authorized).

Although the high presence of collagen in the lower uterine segment reduces bleeding from a hysterotomy and facilitates hysterorrhaphy, the resulting damage appears to be highly favorable to the subsequent development of PAS in this topography of the uterus. The upper portions of the uterine segment, close to the topography of the peritoneal reflection, present notable changes in their thickness, greater supply of growth factors and better blood supply coming from direct and anastomosed branches of the uterine arteries. Therefore, with the internal cervical os as the lower limit, three zones can be identified in the uterine segment: a short upper one, an intermediate one and a lower one, the thinnest of all, with a height of 2-4 cm, located behind and adhered to the bladder wall (Figure 1).^(8)^

Green zone: superior, close to the peritoneal reflection, thicker and vascularized.

Yellow zone: intermediate.

Red zone: lower, the thinnest, 2-4 cm high, behind and attached to the bladder wall.

Hysterotomies are usually performed in the lower uterine segment area (less irrigated), resulting in less fibroblast migration and a poor healing process. Hysterotomies performed in the upper uterine segment bleed more, but are safe and easy to repair and, contrary to the prevailing concept that this area is more prone to rupture, this risk is practically ruled out.^(9)^ Therefore, repairing full-thickness defects and performing hysterotomies in the upper uterine segment area seems to contribute to eliminating healing problems and full-thickness defects. This points to the need to rethink older recommendations and eliminate this risk of PAS associated with cesarean sections with low hysterotomies, particularly those carried out in advanced stages of labor and when fetal extraction is difficult.^(5)^

## What are the general principles that facilitate fetal extraction in cesarean section?

The main strategies for obtaining atraumatic fetal extractions in cesarean sections are the creation of appropriately sized incisions and pharmacological uterine relaxation.^(10)^

Given possible difficulties in fetal extraction, the transverse skin incision should not be less than 15 cm. The abdominal wall opening and hysterotomy must also be of adequate size. The uterine incision must always be longer than 10 cm. The adoption of Maylard's extended laparotomy and transverse segmental hysterotomy with cephalad-caudad blunt expansion should be evaluated. In this, after an incision of the anterior uterine segment performed with the scalpel and forceps, the operator performs the hysterotomy with blunt digital expansion, with the index and middle fingers of one hand pulling towards the uterine body and the same fingers of the other hand expanding in the direction of the cervix. This technique provides a protective and potentially wider opening of the uterine arteries.^(11)^ Also with the intention of preventing vascular and ureteral injuries, and despite the greater risk of uterine rupture in subsequent pregnancies, the option can be to perform extended hysterotomies in inverted T or J. Therefore, when deciding for these incisions, fetal statics and size, the location of the placenta, the presence of leiomyomas, the development of the lower uterine segment and future pregnancy plans must be considered.^(10,11)^ Fetal head extraction should preferably be performed in the occipital (OP) or occiput sacrum (OS) position. Therefore, the fetal head must be rotated to OP or OS using the Geppert maneuver, and released through hysterotomy by its biparietal diameter (9.5 cm), smaller than the occipital frontal diameter (13 cm) of the transverse position varieties.^(12)^

Adequate uterine relaxation can be achieved with an intravenous infusion of 50 μg of nitroglycerin. This dose can be repeated four more times at 60-second intervals until relaxation is adequate. Attention should be paid to maternal hypotension and fetal hypoxia.^(13)^ Other uterolytics, such as beta-agonists (terbutaline, salbutamol) and atosiban can also be used. In patients undergoing general anesthesia, inhalational agents used for anesthetic maintenance, such as sevoflurane, desflurane and isoflurane, also provide dose-dependent uterine relaxation.^(14)^

## What is the pathophysiology and how should the deeply impacted fetal head be managed?

When the entire fetal head occupies the vagina during the expulsion period, the vaginal tissues mold themselves to the fetal head resulting in a "suction" effect similar to that promoted by vacuum extractors cups. The immobility of the cephalic pole on vaginal examination and/or the absence of space between the fetal head and the pubic symphysis, confirmed by the difficulty in introducing the hand that elevates and extracts the fetus through the hysterotomy during the cesarean section, demonstrates the deep insinuation of the fetal head. Impaction occurs in approximately 16% of cesarean sections performed during the expulsion period and is usually associated with a prolongation of the expulsion period and/or unsuccessful attempts at operative vaginal birth.^(15)^

When managing the impacted fetal head, the usual fetal extraction maneuvers are often performed using excessive force with a greater likelihood of maternal and fetal trauma. Lacerations of the lower uterine segment, hematomas in the uterine ligaments and injuries to the uterine vessels, cervix and/or urinary tract are more common. Uterine trauma evolves into postpartum hemorrhage, and the risk of puerperal infection is greater. The newborn can develop serious injuries, such as intracranial hemorrhage, skull fracture, nerve injuries, cervical spine fracture, perinatal asphyxia and even death. When associated with a prolonged expulsion period and/or unsuccessful attempts at operative vaginal birth, it is not always possible to determine if the injuries resulted solely from the maneuvers performed.^(15–17)^

The preparation of the patient and the team must include guidance from the parturient and companions regarding the obstetric challenges linked to the situation, discussion between the obstetrics, anesthesiology and nursing teams, and the development of a fetal extraction plan. Management can be optimized through adequate uterine relaxation, good positioning of the hysterotomy and slow and careful insertion of the hand that manipulates the fetal head. The combined use of the vaginal hand or instruments that elevate the fetal head can provide controlled extraction of the cephalic pole. If an abdominovaginal birth is planned, the parturient must be quickly positioned with the lower limbs elevated, while avoiding contamination of the surgical field at the same time. The time after hysterotomy must be timed, recorded and communicated to those involved in the care.^(4)^

In cesarean sections performed during prolonged expulsion periods, the hysterotomy area is usually larger. Very low hysterotomies should be avoided, as the risk of extending the incision into the vagina is greater, increasing the likelihood of bladder and/or ureteral injury and making surgical repair extremely difficult. Guidelines for managing the impacted fetal head also include: not acting with haste and force, spending a few seconds evaluating the anatomy, even in the face of fetal bradycardia, and not flexing the wrist against the myometrium between the incision and the uterine cervix. After slowly positioning the hand under the fetal head, pressure should be applied towards the maternal abdomen, with the intention to elevate the fetal head and body. Optionally, the assistant can try to move the fetal shoulders towards the mother's head, while the other obstetrician tries to extract the cephalic pole through the hysterotomy. The extraction of the fetal head should only be performed when it is occupying the maternal abdomen, completely detached from the pelvis. In the scenario where there is no space for the hand to penetrate between the fetal head and the pubis, extraction can be obtained by positioning the hand lateral to the fetal head. This strategy is made easier since the transverse diameter of the pelvis is larger than the anteroposterior diameter. Keeping the wrist straight and the arm in the midline, avoiding pressing on the uterine angles, the hand is moved under the fetal face or neck. Sequentially, the fetal head is flexed and elevated towards the mother's head, keeping the arm erect and in the midline, parallel to the mother's body.^(4)^

**Figure 2:**
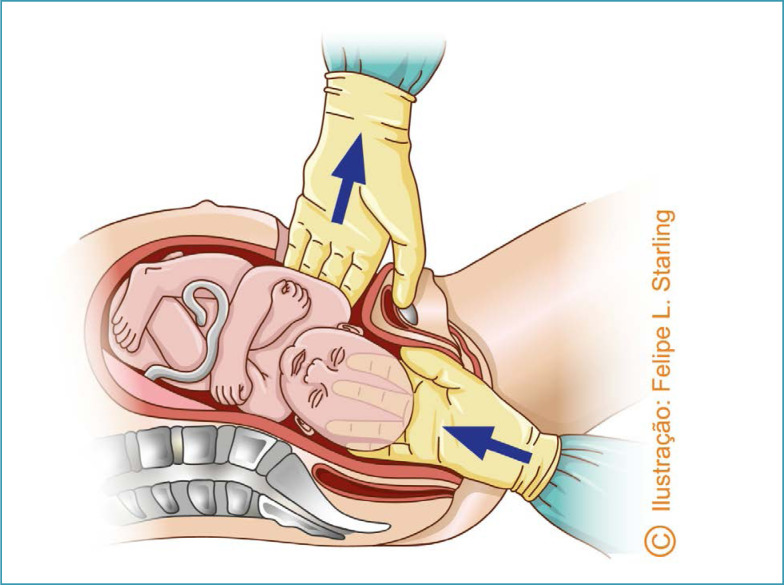
Abdominovaginal delivery (*push method*)

Source: Illustration by Felipe Lage Starling (auhtorized).

Several specific maneuvers can be used for the abdominal delivery of deeply impacted fetuses. In the abdominovaginal delivery (push method), the parturient is positioned with her legs flexed and abducted, and may also be supported in stirrups. After abdominal and vaginal asepsis and antisepsis, an assistant inserts a vaginal hand through the sacral void, grasps the fetal head and moves it superiorly, undoing the impaction and facilitating fetal extraction by the obstetricians performing the cesarean section. The assistant's fingers must be positioned apart, seeking to grasp the largest possible area of the skull, avoiding excessive, potentially traumatic focal pressure. Using the abdominal route, one of the operators simultaneously performs upward traction on the fetal shoulders. Once the fetus is disimpacted, the fetal head is flexed and extracted through the hysterotomy (Figure 2).^(18,19)^ In exceptional situations, this maneuver can be performed by one of the obstetricians performing the cesarean section. While one of the hands is inserted into the vagina to elevate the cephalic pole, the other is kept on the abdomen to prevent its deflection. Immediately after disimpaction, the operator must change gloves and complete the abdominal extraction.^(19,20)^ Although efficient, this maneuver is more associated with prolongation of the hysterotomy, trauma to the uterus and adjacent structures, skull fractures and severe fetal morbidity.^(21,22)^

**Figure 3:**
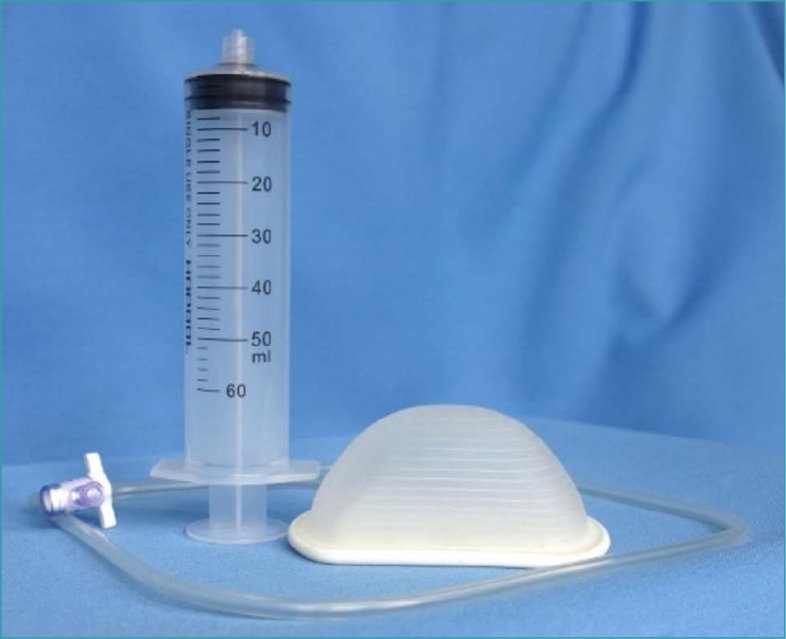
Fetal pillow

Source: Barbieri (2020).^(23)^

When well applied via the combined vaginal route, obstetric levers (or the branch of a forceps) potentially reduce the likelihood of uterine trauma in abdominovaginal delivery. Disimpacting systems such as the fetal pillow have also been used and studied (Figure 3).^(23)^ This is a disposable balloon cephalic elevation device inserted below the fetal head just before the cesarean section. Immediately before laparotomy, the balloon is infused with 180 mL of saline solution, providing an elevation of 2-3 cm of the fetal head. The device is deflated and removed shortly after the cesarean section is complete. Initial evidence from a meta-analysis including heterogeneous and predominantly observational studies indicated that the use of the fetal pillow is associated with a reduction in the time between hysterotomy and delivery, extension of hysterotomy, blood loss, need for blood transfusion, other operative complications and length of hospital stay.^(23)^ Neonatal acidemia, the risk of neonatal sepsis and the need for neonatal intensive care were also lower.^(24)^

The use of disimpacting systems associated with research protocols, clinical regulations and audit systems may be recommended, although improved maternal and neonatal results needs to be investigated through more robust scientific evidence.^(25)^

**Figure 4:**
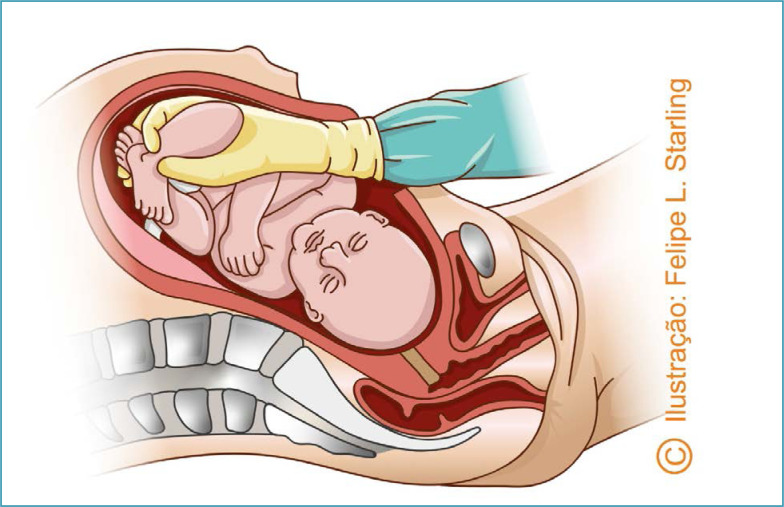
Reverse breech extraction (*pull method*)

Source: Illustration by Felipe Lage Starling (authorized).

**Figure 5:**
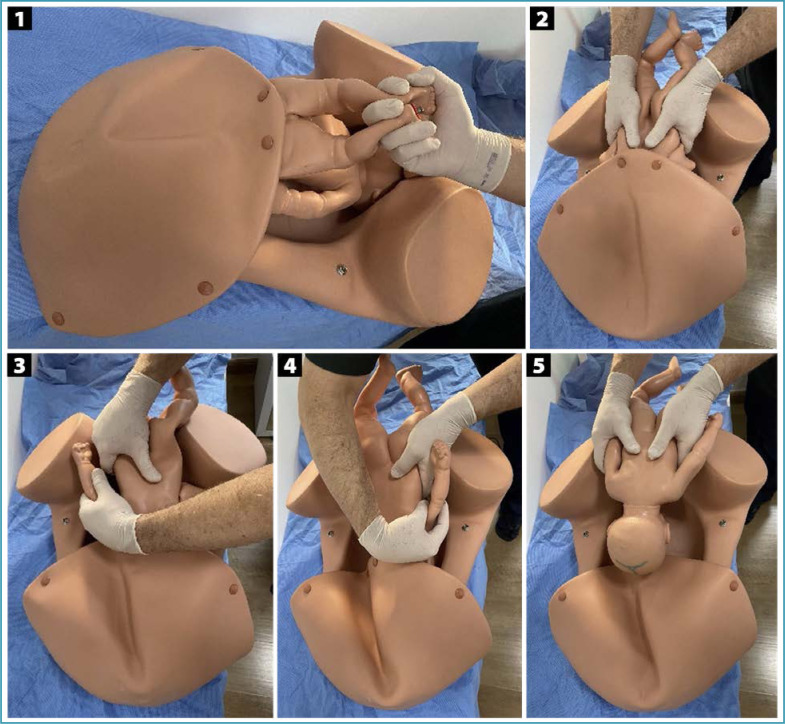
Sequencing of the release of fetal body segments in reverse breech extraction (*pull method*)

Source: Photographic records of the authors.

In reverse breech extraction (pull method) after enlarged hysterotomy, the operator's hand must be inserted towards the uterine fundus. The fetal ankles are grasped and pulled inferiorly. Traction must be applied parallel to the axis of the legs, avoiding fracturing the tibia and/or fibula. After the pelvic pole version, delivery is performed as a classic pelvic extraction by applying the Mauriceau-Smellie-Veit maneuver (Figures 4 and 5).^(26)^ Compared to abdominovaginal delivery (push method), the reverse breech extraction reduces the length of hysterotomy, blood loss, the need for blood transfusion and surgical time.^(27–29)^

1. Delivery of the legs. 2. Delivery of the trunk by flexion of the thoracolumbar region. 3. Delivery of the first shoulder. 4. Delivery of the second shoulder. 5. Delivery of the head.

In the Patwardhan maneuvers described in 1957, reverse breech extraction is preceded and optimized by the release of the upper limbs through hysterotomy, with the operator's hands positioned on the abdomen and pelvis of the fetus. The position of the fetal back determines the sequencing of limb release and the positioning points of the operator's hands and fingers for the reversal of the fetal trunk.^(30,31)^

**Figure 6:**
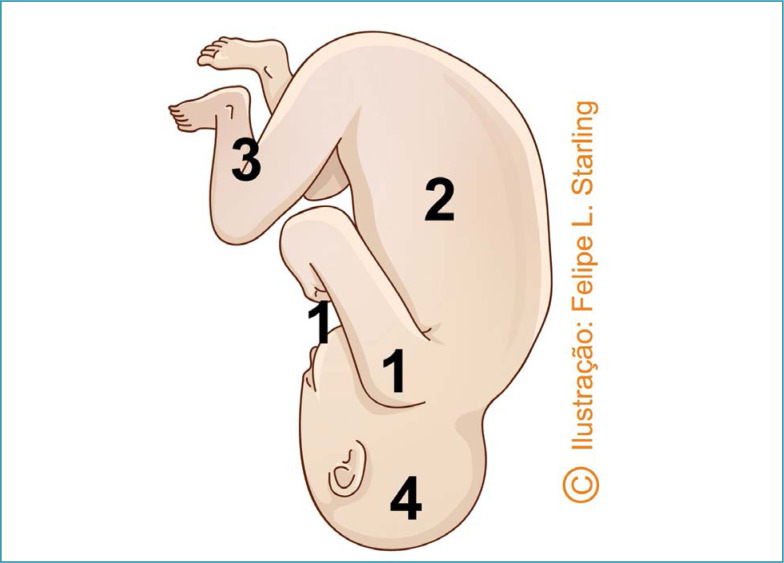
Patwardhan "shoulder first" maneuver for the delivery of fetuses with anterior back

Source: Illustration by Felipe Lage Starling (authorized).

**Figure 7:**
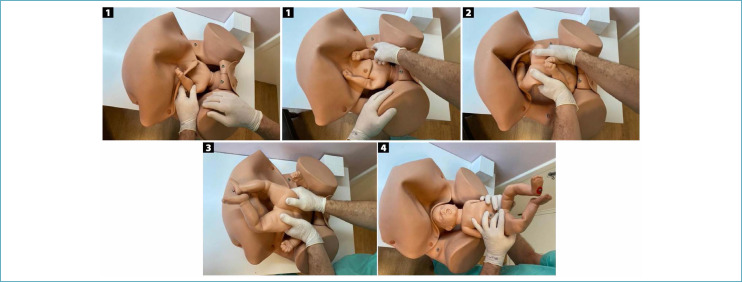
Sequencing of the delivery of fetal body segments in the "shoulder first" Patwardhan maneuver in fetuses with anterior dorsum

Source: Photographic records of the authors.

For deeply impacted fetuses with an anterior back, the recommended Patwardhan maneuver is "shoulder first". This is the most frequent fetal static, usually with anterior oblique position varieties (left occipiut anterior [LOA] and right occiput anterior [ROA]) or OP. The sequencing begins with the delivery of the shoulders through hysterotomy, starting with the most easily accessible arm. After complete delivery of the arms, the operator's hands are positioned bilaterally on the lower part of the fetal trunk, with the support of index fingers on the posterosuperior iliac spines and the other four fingers on the fetal abdomen. The trunk is reversed by a flexion movement with subsequent delivery of the lower limbs and fetal head, without the need for manipulation of these body segments (Figures 6 and 7).^(30,31)^

1. Delivery of the shoulders. 2. Delivery of the trunk by flexion. 3. Delivery of both legs. 4. Delivery of the head.

1. Delivery of the shoulders. 2. Delivery of the trunk by flexion. 3. Delivery of the legs. 4. Delivery of the head.

**Figure 8:**
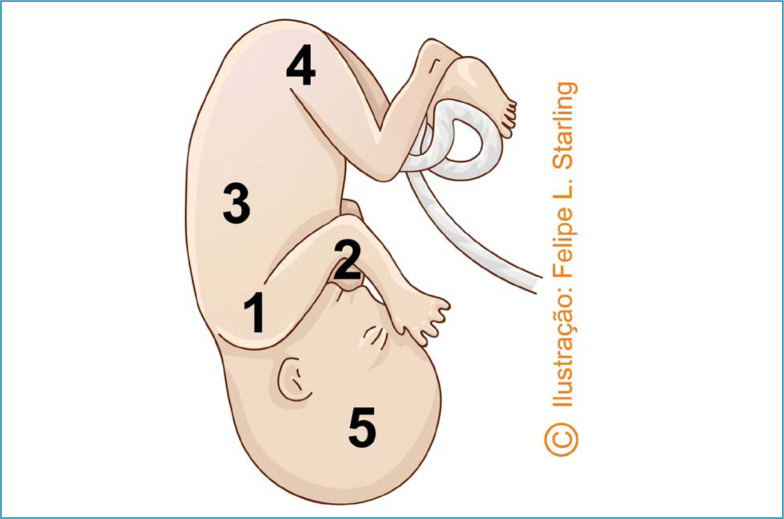
Patwardhan "shoulder first" maneuver for the delivery of fetuses with lateral backs

Source: Illustration by Felipe Lage Starling (authorized).

**Figure 9:**
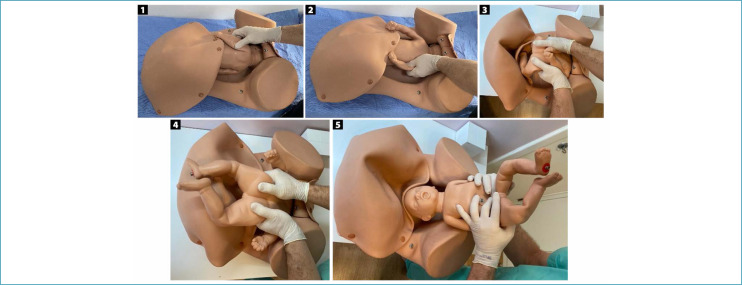
Sequencing of the delivery of fetal body segments in the "shoulder first" Patwardhan maneuver in fetuses with lateral backs

Source: Photographic records of the authors.

In fetuses with lateral dorsum (on the left or right), the recommended Patwardhan maneuver is also "shoulder first". The varieties of position are usually transverse (left occiput transverse [LOT] and right occiput transverse [ROT]). The sequencing begins with the delivery of the shoulders, starting with the anterior arm that will be on the same side of the fetal back and more easily accessible. After delivery of the anterior arm, it is necessary to rotate the fetal trunk for better positioning and delivery of the posterior arm. Next, the trunk will be reversed and released by a flexion movement followed by the delivery of the lower limbs and fetal head (Figures 8 and 9).^(30,31)^

1. Delivery of the anterior shoulder. 2. Delivery of the posterior shoulder. 3. Delivery of the trunk by flexion. 4. Delivery of the legs. 5. Delivery of the head.

1. Delivery of the anterior shoulder. 2. Delivery of the posterior shoulder. 3. Delivery of the trunk by flexion. 4. Delivery of the legs. 5. Delivery of the head.

**Figure 10:**
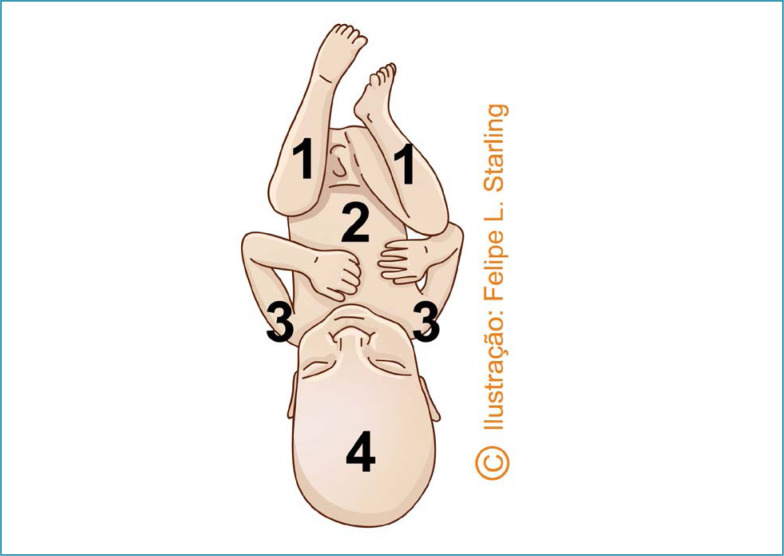
First maneuver proposed by Patwardhan for the delivery of fetuses with posterior dorsum, identical to reverse breech extraction using the pull method

Source: Illustration by Felipe Lage Starling (authorized).

**Figure 11:**
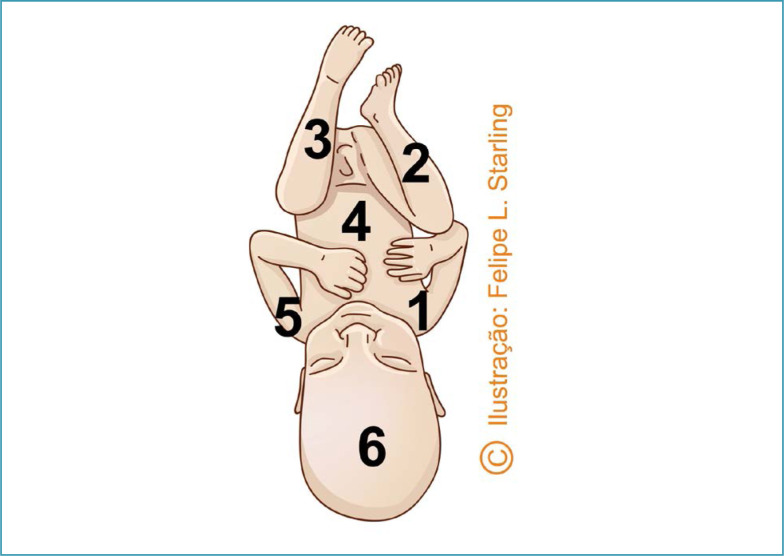
Second Patwardhan maneuver for the delivery of fetuses with posterior dorsum

Source: Illustration by Felipe Lage Starling (authorized).

**Figure 12:**
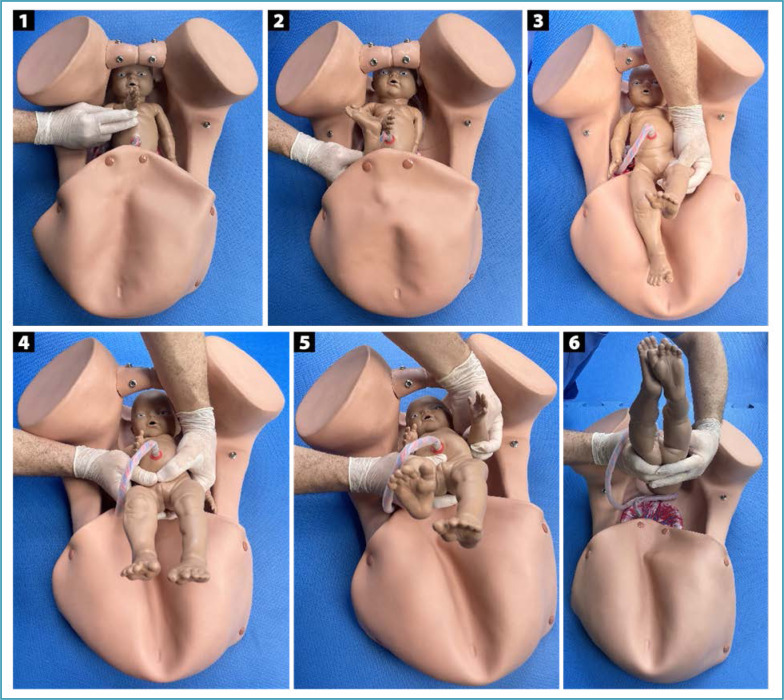
Sequencing of the delivery of fetal body segments in the second Patwardhan maneuver for the delivery of fetuses with posterior dorsum

Source: Photographic records of the authors.

In fetuses with a posterior back, usually in posterior oblique positions (left occiput posterior [LOP] and right occiput posterior [ROP]) or OS, two maneuvers are proposed by Patwardhan. The first is identical to the reverse breech extraction using the pull method, in which the operator's hand inserted into the uterine fundus grasps the ankles and pulls them inferiorly to obtain the pelvic pole version and classic pelvic extraction (Figure 10). In the second maneuver, the sequence begins with the delivery of one of the arms, the most easily accessible. Then, the leg on the same side as the arm that was removed is delivered. Next, the leg on the contralateral side is released. After the delivery of these three limbs, the operator's hands are positioned bilaterally on the lower part of the fetal trunk with the support of index fingers on the fetal abdomen and the other four fingers on each side of the fetal back. The trunk is reversed and released by a flexion movement, followed by the delivery of the arm that was not previously released and the fetal head (Figures 11 and 12).^(30,31)^

1. Delivery of the legs. 2. Delivery of the trunk by flexion. 3. Delivery of the shoulders. 4. Delivery of the head.

1. Delivery of the arm on one side. 2. Delivery of the leg on the same side. 3. Delivery of the opposite leg. 4. Delivery of the trunk by flexion. 5. Delivery of the opposite arm. 6. Delivery of the head.

1. Delivery of the arm on one side. 2. Delivery of the leg on the same side. 3. Delivery of the opposite leg. 4. Delivery of the trunk by flexion. 5. Delivery of the opposite arm. 6. Delivery of the head.

In a meta-analysis of observational studies, prolongation of hysterotomy was more associated with abdominovaginal delivery (push method) than with Patwardhan maneuvers.^(32)^ However, in a more recent meta-analysis comparing abdominovaginal delivery with Patwardhan maneuvers, no differences were found between the techniques regarding the length of the uterine incision, blood transfusion, urinary tract injuries, postpartum hemorrhage, admission to a neonatal intensive care unit and 5-minute Apgar score < 7, thereby making practical recommendations difficult.^(29)^

## What is the pathophysiology and how should the floating fetal head be managed?

The non-insinuated, floating fetal head is defined by its height at or above De Lee's −3 plane. Although in this situation access to the fetal head is easy during cesarean section, the apprehension necessary to perform its traction until hysterotomy is difficult to obtain. In cesarean sections performed on fetuses that are more adjusted to the pelvis, close to the insinuation, when the head is elevated and flexed towards the hysterotomy, uterine forces move it in the same direction, which is the topography of least resistance. On the contrary, in the floating head, uterine forces do not move it through the incision, and the pressure placed on the uterine fundus becomes inefficient, moving it laterally. In this situation, the internal podalic version followed by pelvic extraction or extraction using a vacuum extractor, lever or forceps are the easiest and safest options and the first is usually faster.^(33)^ Pay attention to the contraindication to use of the vacuum extractor before 32 weeks and to its cautious use at 32-36 weeks, since the lower safety limit for gestational age has not yet been established.^(34)^

The version in this scenario differs from the reverse breech extraction (pull method), because, with the fetal head high in the pelvis, the fetus is internally thrown before the extraction of the body segments, similar to the version performed in the vaginal delivery of the second twin with floating cephalic presentation. The procedure is performed by inserting one of the operator's hands deep into the uterus, then grasping one or both feet by the ankle(s) and pulling them through the hysterotomy. Simultaneously, the other hand positioned external to the uterus guides the fetal head towards the uterine fundus.^(33)^

**Figure 13:**
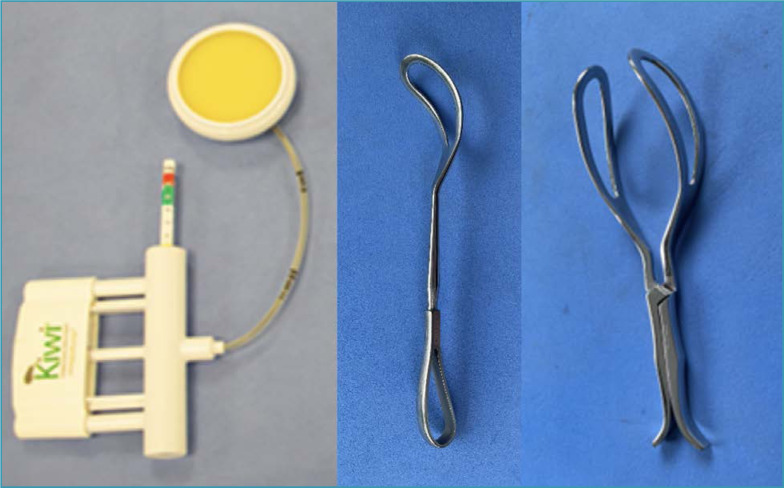
Instruments used in the delivery of fetuses with a floating head

Source: Photographic records of the authors.

Optionally, fetuses with floating heads can be extracted using instruments. After hysterotomy and amniotomy, a vacuum extractor cup, preferably flexible, can be placed at the flexion point of the fetal head. In cesarean sections, vacuum extraction is usually achieved without major difficulties, as there is minimal anatomical resistance through the hysterotomy. Other options include using levers and forceps. Some levers options designed specifically for difficult fetal extraction in cesarean sections are already available in the country. The levers can also be replaced by one of the forceps branches traditionally used in operative vaginal deliveries (Simpson, Kielland). Depending on the obstetrician's desire and experience, forceps can also be used with application of both branches, followed by articulation, assessment of safe grip and traction. There are smaller instruments designed specifically for fetal extraction in cesarean sections, and the Marelli forceps is the most common in our country (Figure 13).^(2,35)^

From left to right: Kiwi OmniCup vacuum extractor, obstetric lever and Marelli forceps.

## Final considerations

The greater prevalence of high-risk pregnancies determining earlier terminations and the evolution of neonatal intensive care, providing greater survival for premature newborns, have increased the prevalence of cesarean sections in fetuses with low birth weight and in anomalous presentations. Childbirth care has evolved contemporaneously with greater safety and use of cesarean sections, which currently have rates above those of scientific recommendations in most countries. At the same time, instrumentation in vaginal birth has reduced drastically, with greater unpreparedness of the new generation of obstetricians, inability of teachers to teach the practice and increasing medical judicialization related to the procedures. This scenario led to an increase in cesarean sections performed in the second stage of labor, also frequently associated with difficulties in fetal extraction. The suggested association of the PAS with low hysterotomies, in particular with cesarean sections performed during the expulsion period of deeply impacted fetuses adds an obstetric risk of high lethality. Therefore, the acquisition of skills and competencies related to childbirth care, the use of forceps and vacuum extractors in the vaginal route of delivery and specific maneuvers for difficult fetal delivery in cesarean sections has become essential in the current process of training obstetricians, justifying the importance of optimizing the study of this topic.

## National Specialized Commission on Obstetric Emergencies of the Brazilian Federation of Gynecology and Obstetrics Associations (Febrasgo)

**President:**

Álvaro Luiz Lage Alves

**Members:**

Gabriel Costa Osanan
Samira El Maerrawi Tebecherane Haddad
Adriana Amorim Francisco
Alexandre Massao Nozaki
Brena Carvalho Pinto de Melo
Breno José Acauan Filho
Carla Betina Andreucci Polido
Eduardo Cordioli
Frederico José Amedée Peret
Gilberto Nagahama
Laíses Braga Vieira
Lucas Barbosa da Silva
Marcelo Guimarães Rodrigues
Rodrigo Dias Nunes
Roxana Knobel
